# Knowledge, attitude and self-efficacy towards palliative care among nurses in Mongolia: A cross-sectional descriptive study

**DOI:** 10.1371/journal.pone.0236390

**Published:** 2020-07-23

**Authors:** Jin Sun Kim, Jinhee Kim, Delgersuren Gelegjamts

**Affiliations:** 1 Department of Nursing, Chosun University, Gwangju, Korea; 2 Graduate School, Chosun University, Gwangju, Korea; 3 Department of Nursing, Mongolian National University, Ulaanbaatar, Mongolia; University of Auckland, NEW ZEALAND

## Abstract

**Background:**

Nurses have a primary role in providing palliative and end-of-life (EOL) care. Their knowledge of EOL care, attitudes toward care of the dying, and palliative care self-efficacy are important in care delivery. Little is known regarding palliative care preparedness among Mongolian nurses. This study examines palliative care knowledge, attitude towards death and dying, and self-efficacy among Mongolian nurses, and examines predictors of self-efficacy.

**Methods:**

A cross-sectional descriptive study was conducted. Participants were 141 nurses employed at the National Cancer Center in Mongolia. Data was collected using a self-administered questionnaire.

**Results:**

The median score for the knowledge of palliative care was 8.0/20. “Psychosocial and spiritual care” was the lowest score on the palliative care knowledge subscale. The mean score for attitude toward care of the dying was 69.1%, indicating positive attitudes. The mean score for the palliative care self-efficacy was 33.8/48. Nurses reported low self-efficacy toward communicating with dying patients and their families, and managing delirium. Palliative care knowledge and duration of experience as an oncology nurse significantly predicted self-efficacy toward palliative care, accounting for 14.0% of the variance.

**Conclusions:**

Palliative education for nurses should address the knowledge gaps in EOL care and focus in increasing palliative care self-efficacy. Considering palliative care knowledge and nursing experience as an oncology nurse were significant predictors of self-efficacy toward palliative care, more effort is needed to fill the knowledge gaps in EOL care among nurses, especially for less experienced nurses.

## Introduction

Palliative care is patient- and family-centered care that improves the quality of life for patients with life-threatening or life-limiting illnesses and their families. The global need for palliative care is increasing, and will continue to do so, considering the growing burden of non-communicable diseases and aging populations [1]. The need for effective palliative care services may become more significant in low and middle-income countries (LMICs), such as Mongolia, given that projections of serious health-related suffering are high in LMICs and fewer resources are available for palliative care [1–3].

Since 2000, palliative care has developed in Mongolia with an establishment of services, policies, and education with support from the International Palliative Care Initiative and Charity [4]. Palliative care standards and pain management guidelines were developed in 2005 and 2012, respectively. During 2010–2015, all hospitals in 21 provinces and all 9 districts of Ulaanbaatar had established palliative beds. Family health centers became responsible for primary palliative care at home. Currently, pain and symptom management are the primary focus, and psychosocial, spiritual and bereavement support are also provided by a multidisciplinary team, including doctors, nurses, and social workers. Although Mongolia has made real progress to integrate palliative care into its health system, services remain limited. There is an unmet need for non-cancer patients, elderly, and children due to a lack of allocated funds and preparedness of health care professionals [3].

Nurses play an important role in providing palliative care [2] and should be prepared to provide quality end-of-lie (EOL) care [5]. Nurses’ palliative care knowledge [5, 6], attitudes toward death and dying [7, 8], and level of self-efficacy [9, 10] can affect the quality of EOL care provided. Previous studies have demonstrated gaps in knowledge [8, 11], moderate to positive attitudes [7, 12], and low-self efficacy [13–15] toward palliative care among nurses. Self-efficacy is an individual’s confidence in skills and perceived ability to perform behavior [16]. It is considered an important predictor of palliative care quality [17, 18]. Therefore, understanding the factors affecting self-efficacy toward palliative care among nurses is necessary to ensure the quality of EOL care. Recently, few studies have explored this construct among nurses [17–19] and identified that palliative care knowledge [17, 18] and attitudes toward EOL care [18, 19] are important predictors of nurses’ self-efficacy toward palliative care.

However, results from previous studies, the majority of which were conducted in developed countries, have limited applicability in LMICS [20, 21]. There are no published studies evaluating the knowledge, attitude, or self-efficacy towards palliative care among Mongolian nurses, and little is known regarding their level of preparedness to deliver palliative care. Although palliative care education is included in the undergraduate nursing curriculum, and post-graduate certificate, diploma, and licensing courses in palliative nursing have been available in Mongolia since 2005, the quality of palliative education in Mongolia may differ from those of high-resource settings.

Moreover, socio-cultural knowledge is important in the understanding of EOL care [22, 23]. Cultural values and beliefs play a role in health care professionals’ attitudes toward death and dying [7, 24, 25], and their level of self-efficacy differed among countries [17]. Traditionally in Mongolia, there is a reluctance to mention death, and its discussion tends to be avoided when in direct contact with the patient [26]. These beliefs and attitudes can also impact the level of palliative education [24].

Therefore, it is necessary to evaluate knowledge, attitudes, and self-efficacy toward EOL care in Mongolian nurses in order to better understand issues that affect their ability to deliver palliative care, and develop educational programs that are appropriate in Mongolian socio-cultural contexts. The purpose of this study was to examine knowledge, attitudes, and self-efficacy toward EOL care among Mongolian oncology nurses, and to identify variables related to self-efficacy. Predictors of self-efficacy toward EOL care were also investigated.

## Methods

### Study design and participants

This was a cross-sectional, descriptive study. Nurses employed at the National Cancer Center (NCC) in U city were recruited for the study. The NCC provides tertiary cancer care, offers palliative care training for health care professionals, and has a palliative care department with 21 beds. A total of 165 nurses with at least three months experience as an oncology nurse, and who understood the purpose of the study and the Mongolian language were potential participants. Questionnaires were distributed to 155 nurses who met inclusion criteria, and 145 were returned. After excluding four participants with incomplete data, 141 participants were included in final analysis.

### Measurements

A demographics form was developed, based on previous studies related to nurses’ palliative care knowledge, attitudes toward care of the dying patient, and self-efficacy toward palliative care. This form was used to collect data and included questions about age, gender, educational level, marital status, religion, position as a nurse, duration of work experience as a nurse and oncology nurse, having a palliative nursing certification, experience of death of a family member or friend, and experience of caring for a dying patient.

#### Knowledge of palliative care

Knowledge of palliative care was defined as the basic palliative and EOL care knowledge necessary to provide quality EOL care for dying patients in all settings. Knowledge was measured using the 20-item Palliative Care Quiz for Nursing (PCQN) [27]. The PCQN consists of three subscales and includes 1) four items on “Philosophy & Principle of Palliative Care,” 2) three items on “Psychosocial and Spiritual Care” in EOL care, and 3) thirteen items on “Management of Pain and Other Symptoms.” Each item was given an answer of “True,” “False,” or “Don’t Know.” Correct answers received a score of 1, and incorrect or “Don’t Know” answers received a score of 0. Individual scores were totaled to produce an overall score, which ranges from 0 to 20. Moreover, percentage of correct answers was also calculated to compare the knowledge level among subscales, which each have a different number of items. Higher scores and percentages indicate knowledge that was more accurate. At the time of development, the internal consistency of the PCQN was 0.78, as measured using the Kuder-Richardson Formula 20 (KR-20) [27].

#### Attitudes toward EOL care

Attitudes toward EOL care was defined as feelings, thoughts, attitudes, and comfort level toward care of the dying patient and their family, and was measured using the Frommelt Attitudes Toward Care of the Dying (FATCOD) Form A [28]. This tool is specifically designed for evaluating nurses, and has an equal number of positively and negatively worded items. Participants are asked to rate their attitudes toward EOL care using a 5-point Likert scale, with negative items reversed for scoring. An overall score is calculated, which ranges from 30 to 150. Higher scores reflect more positive attitudes toward EOL care. The overall score is translated to a percentage score ranging from 0 to 100%, and scores > 65% indicate positive attitudes toward care of the dying [29]. This scale has demonstrated good internal consistency with a Cronbach’s alpha of 0.94 in a previous study [28], and 0.70 in this study.

#### Self-efficacy toward palliative care

Self-efficacy toward palliative care was defined as the perceived capacity to provide palliative care, and was measured using the Palliative Care Self-Efficacy Scale (PCSES), which was developed by Phillips et al. [14]. This 12-item scale has two subscales related to perceived capacity to answer patients’ EOL care concerns, and respond to patients’ EOL symptoms. Participants are asked to rate their perceived capacity to successfully perform each task using a 4-point Likert scale (1 = need further basic instruction; 2 = confident to perform with close supervision/coaching; 3 = confident to perform with minimal consultation; or 4 = confident to perform independently). Possible scores range from 12 to 48, and higher scores indicate a higher perceived capacity toward palliative care. This scale has demonstrated good internal consistency with a Cronbach’s alpha for the entire scale of 0.92, and 0.89 and 0.87 for the subscales, respectively [14]. In this study, the Cronbach’s alpha for the scale was 0.88, and 0.72 and 0.85 for the subscales, respectively.

Permission to use the measurements for this study was obtained from the original developer of each measurement. All measurements were developed in English and previously had not been used with Mongolian subjects. Therefore, a translation and cross-cultural adaption process was necessary to preserve content validity and cultural sensitivity [30]. This process included forward-translation, back-translation, expert panel review, and a pilot test. The English version of the questionnaire was translated into Mongolian by two independent bilingual scholars, and the two translations were compared. The back-translation from Mongolian to English was conducted by two other independent bilingual scholars. Divergent and ambiguous expressions were identified and resolved by a bilingual expert panel. A pilot test with 5 oncology nurses was then carried out. Minor modifications for three items were made to make the questionnaire more understandable for Mongolian nurses. For item 4, adjuvant therapies were explained by adding some examples, such as massage, acupuncture, listening music. The words “bowel regimen” in item 8 were defined by adding the words “laxative treatment.” For item 16, the drug name “Demerol” were explained by adding the commonly used name in Mongolia, “Pethidine.”

### Data collection and ethical considerations

Institutional permission to collect data was obtained after a member of the research team described the study and its purpose to the director of the nursing department. Approval was obtained from the University Institutional Review Board (no. 2-1041055-AB-N-01-2018-31, Chosun University Institutional Review Board) prior to beginning the study. A member of the research team described the study purpose, and measures taken to ensure participant confidentiality and anonymity to potential participants. Written informed consent was obtained from all participants. Participants were also informed that their decision to participate in the study would not affect their employee status.

Each nurse completed a self-administered questionnaire at the hospital, which took approximately 10–15 minutes to complete. Data collection took place between July and August 2018. Upon completion of the questionnaire, each participant received a small gift (water bottle and cup).

### Data analysis

Data were analyzed using SPSS 23.0. Descriptive statistics (frequencies, percent, mean or median, and standard deviation or interquartile range [IQR]) were used to summarize demographic characteristics and to calculate the level of palliative care knowledge, attitudes toward care of the dying patient, and self-efficacy toward palliative care. Kolmogorov-Smirnov tests, histograms, and Q-Q plots were used to check for the assumption of normality in age, duration of working experience as a nurse and oncology nurse, PCQN, FATCOD, and PCSES scores. Since age, duration of working experience as a nurse and an oncology nurse, and PCQN score were not normally distributed, nonparametric presentation and analyses were used to present the scores, including median and IQR. The t-test and one-way analysis of variance were used to determine how self-efficacy toward palliative care varied by participant characteristics. Pearson’s correlation coefficients were used to analyze the relationship between self-efficacy toward palliative care and related continuous variables, including age, duration of working experience as a nurse and an oncology nurse, palliative care knowledge, and attitude toward the care of dying. Multiple regression analysis was conducted to determine predictors of self-efficacy toward palliative care.

## Results

### Characteristics of participants

A total of 141 nurses participated in the study. The median age was 37.5 years (IQR = 26.0–47.0). Participant characteristics were as follows: 98.6% were women; 71.6% had a bachelor’s degree; 62.4% were married; 56.7% were Buddhist; and 86.9% were staff nurses. The median total years of nursing experience was 10.5 (IQR = 3.1–22.0). The median years of oncology nursing experience was 7.3 (IQR = 2.1–15.6). Regarding training and EOL care, 16.3% of participants had completed a three-month training program and earned a palliative nursing subspecialty certification, 63.6% received additional education in palliative and hospice care, 75.2% of participants experienced the death of a family member or friend, and 87.9% had experience in EOL care (Table 1).

**Table 1:** Characteristics of participants (N = 141).

Variable	Category	n (%)	M (IQR)
Age (years)			37.5 (26.0–47.0)
Sex	Female	139 (98.6)	
	Male	2(1.4)	
Educational level	College	38(27.0)	
	Baccalaureate	101(71.6)	
	Master	2(1.4)	
Marital status	Married	88(62.4)	
	Unmarried	41(29.1)	
	Others	12(8.5)	
Religion	Buddhism	80(56.7)	
	Protestant	3(2.1)	
	None	32(22.7)	
	Others	26(18.4)	
Position	Staff nurse	119(86.9)	
	Charge nurse	13(9.5)	
	Head nurse	5(3.6)	
Total experience as a nurse (years)			10.5 (3.1–22.0)
Experience as a oncology nurse (years)			7.3 (2.1–15.6)
Having palliative nursing certification	Yes	23(16.3)	
	No	118(83.7)	
Training experience in hospice and palliative care	Yes	89(63.6)	
	No	51(36.4)	
Experience of death in family or friends	Yes	106(75.2)	
	No	35(24.8)	
Experience of caring for dying patient	Yes	124(87.9)	
	No	17(12.1)	

Abbreviations: M: median; IOQ: Interquartile range.

### Participants’ knowledge on palliative care & hospice

The median PCQN score was 8.0 (IQR = 7–9) out of 20, or 39.5% correct, with a range of 3 to 13. Participants scored the highest on the “Management of Pain and Other Symptoms” subscale, with a mean percentage of correct answer of 43.9%. The mean percentage of correct answers for the “Philosophy & Principle of Palliative Care” subscale was 37.7%. The “Psychosocial and Spiritual Care” subscale had the lowest mean percentage of correct answers at 25.5%. The two questions with the highest percentages of correctly scored answers were: 1) comparison of chronic and acute pain, and 2) bowel regimen for patients on opioids. The two items with the lowest percentages of correctly scored answers were: 1) extent of the disease determines the method of pain treatment, and 2) family members to remain at the bedside until death. Misconceptions regarding long-term morphine use to control pain (due to fear of drug addiction) were reported in 88.7% of participants (Table 2).

**Table 2:** Palliative care knowledge of participants (N = 141).

Item (Correct Answer %)	Correct	Correct	Don’t know
**Total (39.5)**	n (%)	n (%)	n (%)
**Philosophy & principle of palliative care (37.7)**			
1. Palliative care is only appropriate in situations where there is evidence of a downward trajectory or deterioration. (F)	44(31.4)	95(67.9)	1(0.7)
9. The provision of palliative care requires emotional detachment. (F)	1(0.7)	137(97.9)	2(1.4)
12. The philosophy of palliative care is compatible with that of aggressive treatment. (T)	111(79.3)	8(5.67)	21(15.0)
17. The accumulation of losses makes burnout inevitable for those who work in palliative care. (F)	55(39.0)	70(49.6)	16(11.3)
**Psychosocial & spiritual care (25.5)**			
5. It is crucial for family members to remain at the bedside until death occurs. (F)	5(3.5)	136(96.5)	-
11. Men generally reconcile their grief more quickly than women. (F)	60(42.6)	38(27.0)	43(30.5)
19. The loss of a distant relationship is easier to resolve than the loss of one that is close or intimate. (F)	43(30.7)	46(32.9)	51(36.4)
**Management of pain and other symptoms (43.9)**			
1. Morphine is the standard used to compare the analgesic effect of other opioids. (T)	25(18.)	100(77.7)	6(4.3)
2. The extent of the disease determines the method of pain treatment. (F)	1(0.7)	140(99.3)	-
3. Adjuvant therapies (e.g., massage, acupuncture, listening music) are important in managing pain. (T)	109(78.4)	13(9.4)	17(12.2)
6. During the last days of life, drowsiness associated with electrolyte imbalance may decrease the need for sedation. (T)	73(52.5)	39(28.1)	27(19.4)
7. Drug addiction is a major problem when morphine is used on a long-term basis for the management of pain. (F)	10(7.1)	125(88.7)	6(4.3)
8. Individuals who are taking opioids should also follow a bowel regime (laxative treatment). (T)	124(89.2)	6(4.3)	9(6.5)
10. During the terminal stages of an illness, drugs that can cause respiratory depression are appropriate for the treatment of severe dyspnea. (T)	18(12.9)	102(72.9)	20(14.3)
13. The use of placebos is appropriate in the treatment of some types of pain. (F)	5(3.5)	129(91.5)	7(5.0)
14. High dose codeine causes more nausea and vomiting than morphine. (T)	79(57.2)	26(18.8)	33(23.9)
15. Suffering and physical pain are identical. (F)	47(34.1)	79(57.2)	12(8.7)
16. Demerol (Pethidine) is not an effective analgesic for the control of chronic pain. (T)	82(59.4)	21(15.2)	35(25.4)
18. Manifestations of chronic pain are different from those of acute pain. (T)	128(91.4)	3(2.1)	9(6.4)
20. Pain threshold is lowered by fatigue or anxiety. (T)	97(68.8)	27(29.1)	17(12.1)

Abbreviations: T = true; F = false.

### Attitude toward care of the dying

The mean FATCOD score as a percentage of the total score was 69.1%, with 77.2% of participants being classified as having a positive attitude toward care of a dying patient. The three highest scoring positive attitudes were: “Giving care to the dying person is a worthwhile experience,” “Nursing care should extend to the family of the dying person,” and “Families need emotional support to accept the behavior changes of the dying person.” The three highest scoring negative attitudes were as follows: “I would be upset when the dying person I was caring for gave up hope of getting better,” “When a patient asks ‘Nurse am I dying?’ I think it is best to change the subject to something cheerful,” and “I would be uncomfortable talking about impending death with the dying person” (Table 3).

**Table 3:** Participants’ attitude towards care of the dying (N = 141).

Item	M (SD)
Giving nursing care to the dying person is a worthwhile learning experience.	4.2 (0.7)
Death is not the worst thing that can happen to a person.	3.5 (1.0)
I would be uncomfortable talking about impending death with the dying person.	2.6 (1.0)
Nursing care for the patient's family should continue throughout the period of grief and bereavement.	3.6 (0.9)
I would not want to be assigned to care for a dying person.	3.9 (1.0)
The nurse should not be the one to talk about death with the dying person.	2.9 (1.3)
The length of time required to give nursing care to a dying person would frustrate me.	3.5 (0.9)
I would be upset when the dying person I was caring for gave up hope of getting better.	2.4 (0.8)
It is difficult to form a close relationship with the family of a dying person.	2.9 (1.0)
There are times when death is welcomed by the dying person.	3.8 (0.7)
When a patient asks, "Nurse am I dying?", I think it is best to change the subject to something cheerful.	2.6 (0.9)
The family should be involved in the physical care of the dying person.	2.7 (0.9)
I would hope the person I'm caring for dies when I am not present.	2.7 (1.0)
I am afraid to become friends with a dying person.	3.1 (1.0)
I would feel like running away when the person actually died.	3.7 (0.9)
Families need emotional support to accept the behavior changes of the dying person.	4.1(0.6)
As a patient nears death, the nurse should withdraw from his/her involvement with the patient.	3.8 (1.0)
Families should be concerned about helping their dying member make the best of his/her remaining life.	4.0 (0.6)
The dying person should not be allowed to make decisions about his/her physical care.	3.4 (1.0)
Families should maintain as normal an environment as possible for their dying member.	2.6 (1.0)
It is beneficial for the dying person to verbalize his/or feelings.	4.1 (0.6)
Nursing care should extend to the family of the dying person.	4.2 (0.6)
Nurses should permit dying persons to have flexible visiting schedules.	4.1 (0.7)
The dying person and his/her family should be the in-charge decision makers.	4.1 (0.7)
Addiction to pain relieving medication should not be a nursing concern when dealing with a dying person.	3.0 (1.2)
I would be uncomfortable if I entered the room of a terminally ill person and found him/her crying.	2.7 (0.9)
Dying persons should be given honest answers about their condition.	3.3 (0.9)
Educating families about death and dying is not a nursing responsibility.	3.1 (1.7)
Family members who stay close to a dying person often interfere with the professionals' job with the patient.	3.1 (0.7)
It is possible for nurses to help patients prepare for death.	3.5 (1.0)
**Total score**	103.7 (8.1)
**Mean percentage of total score**	69.1 (5.4)

### Self-efficacy toward palliative care

The mean score for the PCSES was 33.8 out of 48, ranging from 18 to 48. The mean score for the “Perceived capability to answer patient’s EOL care concerns” subscale was 17.0, and the mean score for the “Perceived capability to respond to patient’s EOL symptoms” subscale was 16.9. The two items with the highest self-efficacy scores were, “Answering queries about the effects of concern medications” and “Reacting to and coping with reports of constipation.” The two items with the lowest self-efficacy scores were, “Discussing patient’s wishes for after their death” and “Reacting to and coping with terminal delirium” (Table 4).

**Table 4:** Participants’ self-efficacy toward palliative care (N = 141).

Item	M (SD)
**Perceived capability to answer end-of-life care concerns**	
Answering patients questions about the dying process	2.7 (1.0)
Supporting the patient or family member when they become upset	2.8 (1.0)
Informing people of the support services available	2.9 (1.0)
Discussing different environmental options (e.g., hospital, home, family)	3.1 (0.9)
Discussing patient’s wishes for after their death	2.2 (1.1)
Answering queries about the effects of certain medications	3.3 (0.8)
Subtotal	17.0 (3.7)
**Perceived capability to respond to patient’s end-of-life symptoms**	
Reacting to reports of pain from the patient	3.1 (0.9)
Reacting to and coping with terminal delirium	2.4 (1.1)
Reacting to and coping with terminal dyspnea (breathlessness)	2.6 (1.1)
Reacting to and coping with nausea / vomiting	3.1 (1.0)
Reacting to and coping with reports of constipation	3.1 (0.9)
Reacting to and coping with limited patient decision-making capacity	2.4 (1.1)
Subtotal	16.9 (4.6)
Total	33.8 (7.7)

### Differences in self-efficacy toward palliative care by participant’s characteristics

The differences between mean scores for self-efficacy toward palliative care by participant characteristics (for categorical variables) were analyzed using t-tests or ANOVAs. As presented in Table 5, there were no significant differences in self-efficacy toward palliative care by participant characteristics. Although nurses who received a palliative certification course or palliative care training course reported higher self-efficacy than their counterparts, there were no significant differences between groups. Nurses who had experienced death of a family member or friend reported higher self-efficacy toward palliative care, and nurses who had experience caring for dying patients reported lower self-efficacy toward palliative care than their counterparts, but there were no significant differences between groups.

**Table 5:** Differences of self-efficacy toward palliative care by participants’ characteristics (N = 141).

Variable	Category	M (SD)	t or F	*p*
Sex	Female	33.9 (7.6)	-0.798	.426
	Male	29.5 (16.3)		
Educational Level	College	34.6 (8.2)	1.132	.325
	BSN	33.5 (7.5)		
	Master	44.0 (7.7)		
Marital Status	Married	34.0 (7.6)	0.801	.451
	Unmarried	32.9 (8.0)		
	Others	36.0 (7.8)		
Religion	Buddhism	34.4 (8.0)	0.451	.717
	Protestant	30.7 (4.0)		
	None	33.0 (7.2)		
	Others	33.4 (8.0)		
Position	Staff Nurse	33.2 (7.4)	2.623	.076
	Charge Nurse	33.8 (8.5)		
	Head Nurse	38.5 (6.6)		
Having palliative nursing certification	Yes	36.7 (7.2)	1.967	.051
	No	33.3 (7.7)		
Training experience in hospice and palliative care	Yes	34.6 (7.8)	1.219	.225
	No	32.9 (7.2)		
Experience of death in family or friends	Yes	34.1 (7.6)	0.768	.444
	No	33.0 (8.2)		
Experience of caring for dying patient	Yes	33.8 (7.9)	-0.192	.848
	No	34.2 (6.1)		

### Self-efficacy toward palliative care and related variables

Pearson correlation analysis was used to determine the correlates of self-efficacy toward palliative care for continuous variables, such as age, working experience as a nurse and an oncology nurse, palliative care knowledge, and attitude towards the care of dying were analyzed using. There was a significant positive correlation between self–efficacy toward palliative care and knowledge toward EOL care (r = .23, *p* = .013), duration of experience as a nurse (r = .23, *p* = .007), duration of experience as an oncology nurse (r = .25, *p* = .004), and participant age (r = .202, *p* = .019).

## Predictors of self-efficacy toward palliative care

Based on the results of univariate analyses, participant age, duration of experience as a nurse, duration of experience as an oncology nurse, and knowledge were entered in the regression model. Assumptions to conduct multiple regression analysis were checked including linearity, normally distributed errors, independent errors (Durbin-Watson = 1.592), homoscedasticity, and multicollinearity. However, duration of experience as a nurse (tolerance = .094, VIF = 10.626) and participant age (tolerance = .151, VIF = 6.604) were deleted in the regression model due to multicollinearity between independent variables. Duration of experience as an oncology nurse and knowledge were significant predictors of variance in self-efficacy toward palliative care, accounting for 14.0% of the variance (F = 9.456, *p* < .001). Duration of experience as an oncology nurse was the most significant variable in predicting self-efficacy toward palliative care (Table 6).

**Table 6:** Predictors of participants’ self-efficacy toward palliative care (N = 141).

Predictors	B	SE	β	t (*p*)	*R*^2^ (adj*R*^2^)	F (*p*)
Constant	26.262	2.546		10.313 (< .001)	.140 (.125)	9.456 (< .001)
Duration of working experience as an oncology nurse	.019	.005	.300	3.447 (.001)		
Knowledge	.670	.311	.187	2.152 (.033)		

## Discussion

This study is the first to evaluate the preparedness of palliative care among Mongolian nurses. Nurses in this study demonstrated insufficient knowledge of palliative care, reported positive attitudes toward palliative care, and perceived a lack of confidence regarding the ability to discuss psychological and spiritual aspects with patients and their families.

The median PCQN score in this study was lower than in previous studies conducted in other countries, including Jordan, India, Korea, Australia, Greece, Ireland, and Spain; these scores ranged from 8.3 to 12.5 [8, 31–35]. Only one study conducted in Iran [36] reported a lower PCQN score than this study at 7.6. For three conceptual subcategories of PCQN, the highest percentage of correct answers was for “management of pain and other symptoms”, and the lowest percentage of correct answers was for “psychosocial and spiritual care,” which is consistent with previous studies [32, 34, 36]. It may reflect current focus of EOL care in Mongolia. However, palliative care is a holistic and comprehensive approach, and psychological and spiritual care is an important aspect of EOL care. Nurses should be competent in providing psychological and spiritual care [37]. Additional effort is needed to fill the knowledge gaps in psychological and spiritual care among Mongolian nurses.

In this study, 96.5% of participants scored the PCQN item “It is crucial for family members to remain at the bedside until death occurs” as “True,” in contrast to the PCQN developer’s answer of False. This result is consistent with a Korean study [38] that found 100% of nurses reported as False. Culturally sensitive interpretation is needed for this PCQN item. A Mongolian study [39] demonstrated that 88% of palliative patients prefer to spend their last days of life at home with family. In Asian cultures, nurses as well as patients and family members consider family presence at the moment of death and dying to be a “Good Death.” This concept in the perspective of Mongolian culture should be further defined and accommodated for EOL care.

Moreover, 88.7% of nurses in this study were reluctant to use morphine due to concerns of drug addiction. These results are consistent with those reported by Davaasuren [40], which found that 70% of family doctors in Mongolia, who are mainly responsible for primary palliative care at home, have morphine phobia. Moreover, 78.4% of nurses in this study answered that ‘adjuvant therapies are important in managing pain,’ which may reflect wide acceptance of complementary and alternative medicine for pain management among Mongolians [41]. Previous studies demonstrated that multiple factors may relate to the reluctant use of opioids, including inadequate training of health care professionals, cultural and attitudinal barriers, financial cost, and national regulatory controls related to concerns about abuse and dependence [20, 23]. Further studies are needed to clarify the misconception of opioids and barriers to use of opioids among Mongolian health care professionals.

Previous studies have reported that EOL care education programs improve EOL care knowledge among nurses [5, 6, 42]. Considering knowledge was a significant predictor of self-efficacy toward palliative care, more efforts are needed to improve the quality of palliative care education. Further research is necessary to analyze the contents of palliative care education in Mongolia. Palliative care education can be improved by following recommendations from the End-of-Life Nursing Education Consortium. However, some modifications or alternative palliative education models tailored to health care systems, and to societal and cultural needs in Mongolia may be more important. Mongolia is a large country with a small, widely dispersed population. To ensure the quality of palliative care education in remote or rural areas, development of an online course may be helpful and train-the-trainer programs in limited resources of Mongolia are recommended.

The mean FATCOD score as a percentage of the total score in this study was 69.1%, which is lower than 86.0% among oncology nurses in the United States [43], 79.3% among non-palliative care nurses in Australia [7], and 83.8% among Japanese oncology nurses [12]. Nevertheless, the overall positive attitude toward care of dying in our study is somewhat encouraging and may reflect the earlier acceptance of the national palliative care system and education in Mongolia compare to other LMICs [3].

Effective communication skills with dying patients and their families are critical to ensure the quality EOL care [5]. In this study, scores for communication between the nurse and the dying patient and family were low compared to other items on the FATCOD. Moreover, the lowest self-efficacy item in this study was “Discussing patient’s wishes for after their death.” Some possible explanations may exist for these results. First, it may relate to Mongolian culture. Asian cultures traditionally tend toward reluctance in discussing death with patients [24, 26, 44]. Although Mongolian nurses learned that patients have a right to know about their illness and prognosis, they may still feel uncomfortable disclosing bad news in their cultural context. Therefore, skills that help nurses communicate with dying patients and their families should be culturally sensitive and family-centered approach.

Another possible explanation may relate to the lack of EOL communication content in palliative care education in Mongolia. Therefore, education that addresses communication with dying patients and their families can help improve both attitudes and self-efficacy among nurses providing palliative care [5, 45]. Another possible explanation may relate to communication training strategies in Mongolia, which mainly consist of lecture and discussion formats. However, these strategies may not be enough to increase EOL communication. Previous studies identified that small groups, interactive exercises, and role-playing can be more effective training strategies to improve EOL communication [10]. More effective training strategies need to be adapted in EOL communication education.

The mean score of total PCSES was 33.8 out of 48, indicating that nurses need support in providing palliative care. This result is consistent with previous studies that demonstrated how nurses who attended palliative care education intervention had increased self-efficacy compared to control groups [13, 17]. Although delirium is a common neuropsychiatric disorder in patients receiving palliative care [46, 47], self-efficacy related to reacting to and coping with terminal delirium was low in this study. This finding is consistent with a Japanese study [15], which reported difficulty in managing delirium as one of the barriers to the confidence of nurses. Previous studies identified that nurses with more knowledge regarding delirium reported higher levels of self-efficacy and better palliative care practice [47, 48].

In this study, the significant predictors of self-efficacy toward palliative care were palliative care knowledge and duration of experience as an oncology nurse. These findings are consistent with previous studies [17–19]. An Indonesian study [18] identified that nurses’ palliative care knowledge was one of the most powerful factors of their self-efficacy. Moreover, a study conducted with care staff in long-term care facilities in six European countries [17] identified that working in direct care for >10 years was a significant factor of higher levels of self-efficacy. Previous studies suggested that palliative care education could improve the self-efficacy of healthcare providers [5, 17]. Therefore, palliative care education should include communication skills and delirium management strategies to increase palliative care self-efficacy, especially for less experienced nurses.

There are limitations to this study that should be considered when generalizing these findings. This was a cross-sectional study from one cancer center in a city; thereby, it is important to consider the generalizability of these findings when applied to other populations and study groups. Nevertheless, some findings may be relevant to other LMICs or Asian countries, which have similar socio-cultural context. The questionnaires used in this study were developed by western culture and not modified to consider diverse cultural views toward death. Therefore, a more culturally sensitive scale to measure Mongolian nurses’ EOL care needs to be developed. Social desirability bias may have inflated the positive attitudes toward care of the dying in response to perceived expectations regarding nurses, although the self-administered questionnaire and the anonymity afforded respondents should have kept this to a minimum.

## Conclusion

Our results reflects that nurses have positive attitudes toward palliative care however lack of confidence in providing palliative care and limited knowledge of palliative care. Palliative education and training for nurses should address the knowledge gaps in psychological and spiritual aspects of EOL care and focus in increasing palliative care self-efficacy. Considering palliative care knowledge and nursing experience as an oncology nurse were significant predictors of self-efficacy toward palliative care, more effort is needed to fill the knowledge gaps of EOL care among nurses, especially for less experienced nurses.

## Supporting information

S1 File Questionnaire-permission. (PDF)
