# Supplementary material for: Knowledge, attitude and self-efficacy towards palliative care among nurses in Mongolia: A cross-sectional descriptive study
Source: PLoS One. 2020 Jul 23;15(7):e0236390. doi: 10.1371/journal.pone.0236390 (PMC7377484; doi:10.1371/journal.pone.0236390)
Supplement: S1 File — (PDF) [file pone.0236390.s001.pdf]

Re: The Palliative Quiz for Nursing (PCQN) Permission

fbourbon@uottawa.ca
보낸 날짜: 2018년 12월 3일 월요일 오후 9:51
받는 사람: 김진선(간호학과)

>Hello Dr.Jin Sun Kim. Yes you can use the PCQN. Please just site the source of the tool. I have looked after Dr. Ross correspondence for many years. Best wishes on the research of your doctoral student. Frances

Dear Dr. Fothergill-Bourbonnais
>
>
>
> I am a Korean researcher. I and my Mongolian doctoral student who is
> intersted in palliative care in Mongolia want to use " The Palliative Quiz
> for Nursing (PCQN)" cited in
>
>
>
> Ross, M., McDonald, B., & McGuiness, J. (1996). The Palliative Quiz
> for Nursing (PCQN): development of an instrument to measure nurses
> knowledge of palliative care. Journal of Advanced Nursing, 23,
> 126-137.
>
>
>
> We try to search Dr. Margaret M. Ross's contact information, we found your
> information from previous researchers who alos use the PCQN.
>
>
>
> We will conduct a study to identify Mongolian cancer nurses' knowledge,
> attitudes, and self-efficacy toward palliative care.
>
>
>
> We will going to use this scale after translation to Mongolian language.
>
>
>
> Can you give us a permission to use this scale to us for our study?
>
>
>
> We look forward to hear good news from you.
>
>
>
> Thank you.
>
>
>
>
>
> Jin Sun Kim, PhD, RN
>
> Professor
>
> Department of Nursing, Chosun University,
>
> 309 Pilmun-daero, Dong-gu, Gwangju, 501-759, South Korea
> Office:+82-62-230-6327
> Fax:+82-62-230-6329
>

Re: 회신: Frommelt Attitude Toward Care of the Dying Scale Permission

Katherine Frommelt [kay.frommelt@gmail.com]
보낸 날짜: 2018년 12월 13일 목요일 오전 11:02
받는 사람: 김진선(간호학과)

Dear Jinsun,

I am hereby giving you permission to translate the FATCOD, Form A, and to use it in your study.
Best of luck with your research.

Katherine H Murray Frommelt, PhD, RN, PDE, CGC, FT

Sent from my iPad

On Dec 12, 2018, at 7:06 PM, <jinsun@Chosun.ac.kr> <jinsun@Chosun.ac.kr> wrote:

Dear Professor Frommelt

I am a Korean researcher. I and my Mongolian doctoral student who is intersted in palliative care in Mongolia want to use" Frommelt Attitude Toward Care of the Dying Scale" cited in

Frommelt, K. H. M. (1991). The effects of death education on nurses' attitudes toward caring for terminally ill persons and their families. American Journal of Hospice and Palliative Medicine, 8(5), 37-43.

We will conduct a study to identify Mongolian cancer nurses' knowledge, attitudes, and self-efficacy toward palliative care.

On December 1, I contacted you and you asked about which version we will going to use. We will going to use this scale (Form A) after translation to Mongolian language.

Can you give us a permission to translate this scale to us for our study?

We look forward to hear good news from you.

Thank you.

Jin Sun Kim, PhD, RN
Professor
Department of Nursing, Chosun University,
309 Pilmun-daero, Dong-gu, Gwangju, 501-759, South Korea
Office:+82-62-230-6327
Fax:+82-62-230-6329

보낸 사람: Katherine Frommelt [kay.frommelt@gmail.com]
보낸 날짜: 2018년 12월 1일 토요일 오전 11:44
받는 사람: 김진선(간호학과)
제목: Re: Frommelt Attitude Toward Care of the Dying Scale Permission

Dear Jin Sun

Do you want to use the original form of the tool, which I refer to as Form A, and which was designed for use by nurses only, or the revised FATCOD Form B, which was revised so that it could be used by persons from any discipline, including nurses? Form B also has more demographic questions.

Please advise

Katherine H Murray Frommelt, PhD, RN, PDE, CGC, FT

Sent from my iPad

On Nov 30, 2018, at 8:29 PM, <jinsun@Chosun.ac.kr> <jinsun@Chosun.ac.kr> wrote:

Dear Professor Frommelt

I am a Korean researcher. I and my Mongolian doctoral student who is intersted in palliative care in Mongolia want to use" Frommelt Attitude Toward Care of the Dying Scale" cited in

Frommelt, K. H. M. (1991). The effects of death education on nurses' attitudes toward caring for terminally ill persons and their families. American Journal of Hospice and Palliative Medicine, 8(5), 37-43.

We will conduct a study to identify Mongolian cancer nurses' knowledge, attitudes, and self-efficacy toward palliative care.

We will going to use this scale after translation to Mongolian language.

Can you give us a permission to translate this scale to us for our study?

We look forward to hear good news from you.

Thank you.

Jin Sun Kim, PhD, RN
Professor
Department of Nursing, Chosun University,
309 Pilmun-daero, Dong-gu, Gwangju, 501-759, South Korea
Office:+82-62-230-6327
Fax:+82-62-230-6329

RE: Palliative Care Self-Efficacy Scale

Jane Phillips [Jane.Phillips@uts.edu.au]
보낸 날짜: 2018년 12월 13일 목요일 오후 2:54
받는 사람: 김진선(간호학과)
첨부 파일: PC_Self-Efficacy Scale_PHI~1.pdf (19 KB)

Dear Dr Kim,

Thank you for your inquiry – very happy for you to translate this into Mongolian, just please cite the source.

I have attached the original survey.

Best wishes with your study and look forward to reading the results in an English peer-reviewed journal.

Kind regards
Jane

Jane Phillips, RN PhD, FACN
Professor Palliative Nursing,
Director IMPACCT
IMPACCT – Improving Palliative,
Aged and Chronic Care through
Clinical Research and Translation

Faculty of Health
T. +61 (02) 9514 4862 M. +61 (0)
4111 00617
Level 3, 235 Jones St. Ultimo NSW
2007
PO Box 123. Broadway NSW 2007
Australia
uts.edu.au/impacct

EA Cathy Lambert
T +61 (02) 9514
4862 **E** catherine.lambert@uts.edu.au

We acknowledge the Traditional Custodians of the lands on which we work and live, and pay our respects to Elders past, present and future.

From: jinsun@Chosun.ac.kr <jinsun@Chosun.ac.kr>
Sent: Thursday, 13 December 2018 12:09 PM
To: Jane Phillips <Jane.Phillips@uts.edu.au>
Subject: Palliative Care Self-Efficacy Scale

Dear Professor Jane Phillips

I am a Korean researcher. I and my Mongolian doctoral student who is intersted in palliative care in Mongolia want to use Palliative Care Self-efficacy Scale cited in

Phillips, J., Salamonson, Y., & Davidson, P. M. (2011). An instrument to assess nurses' and care assistants' self-efficacy to provide a palliative approach to older people in residential aged care: A validation study. International Journal of Nursing Studies, 48(9), 1096-1100.

We will conduct a study to identify Mongolian cancer nurses' knowledge, attitudes, and self-efficacy toward palliative care.

We will going to use this scale after translation to Mongolian language.

Can you give us a permission to translate this scale to us for our study?

We look forward to hear good news from you.

Thank you.

Jin Sun Kim, PhD, RN
Professor
Department of Nursing, Chosun University,
309 Pilmun-daero, Dong-gu, Gwangju, 501-759, South Korea
Office:+82-62-230-6327
Fax:+82-62-230-6329

UTS CRICOS Provider Code: 00099F DISCLAIMER: This email message and any accompanying attachments may contain confidential information. If you are not the intended recipient, do not read, use, disseminate, distribute or copy this message or attachments. If you have received this message in error, please notify the sender immediately and delete this message. Any views expressed in this message are those of the individual sender, except where the sender expressly, and with authority, states them to be the views of the University of Technology Sydney. Before opening any attachments, please check them for viruses and defects. Think. Green. Do. Please consider the environment before printing this email.
